# Case Report: Trapdoor procedure combined with arthrodiastasis treating chondroblastoma of the femoral head in adolescents

**DOI:** 10.3389/fped.2025.1668112

**Published:** 2025-11-06

**Authors:** Yuyin Xie, Can Liu, Yifan Chen, Yangfei Yi, Zhongwen Tang, Jie Wen, Sheng Xiao

**Affiliations:** 1 Department of Pediatric Orthopedics, Hunan Provincial People’s Hospital, The First Affiliated Hospital of Hunan Normal University, Changsha, China; 2 Department of Anatomy, Hunan Normal University School of Medicine, Changsha, Hunan, China

**Keywords:** chondroblastoma, femoral head, arthrodiastasis, trapdoor procedure, treatment

## Abstract

Chondroblastoma is a rare benign bone tumor that typically arises in the epiphysis. The primary treatment strategy involves complete curettage of the lesion. However, there is limited literature regarding the management of chondroblastoma specifically located in the femoral head. Following simple curettage alone, the femoral head is prone to collapse due to injury to the growth plate and the effects of weight-bearing stress. Arthrodiastasis can provide a conducive environment for healing and remodeling of the necrotic or grafted femoral head by maintaining continuous distraction of the hip joint space and reducing mechanical pressure. Analogous to the use of skeletal traction in fracture management, sustained arthrodiastasis can effectively counteract compressive forces exerted by muscular contraction on the femoral head. We present the case of a 15-year-old patient with a chondroblastoma localized in the right femoral head who demonstrated favorable clinical and radiographic outcomes following Trapdoor procedure combined with Arthrodiastasis treatment.

## Introduction

Chondroblastoma is a rare benign bone tumor. As the primary treatment modality for this condition, curettage with bone grafting has been extensively employed in the management of bone tumors and has demonstrated favorable therapeutic outcomes (1). However, in pediatric patients with lesions located in the proximal femur, this approach may result in femoral head collapse, which can compromise clinical outcomes. Arthrodiastasis has been predominantly utilized for Legg-Calvé-Perthes disease and avascular necrosis of the femoral head, while its application in the treatment of bone tumors remains relatively uncommon. We report a case of adolescent with chondroblastoma localized in the proximal femur who underwent Trapdoor procedure combined with Arthrodiastasis treatment, resulting in satisfactory clinical and radiographic outcomes.

## Case presentation

A 15-year-old Chinese male patient was admitted to the hospital with a chief complaint of “right hip joint pain and limping lasting for more than 3 months.” The pediatric patient is from Hunan Province, China. Prior to the visit, there was no clear predisposing cause or history of trauma, no previous surgical history, and no relevant medical conditions or treatment history. Physical examination revealed a painful limping gait, mild swelling in the right hip region, asymmetry of the bilateral inguinal folds, localized anterior tenderness over the right hip, and absence of percussion tenderness at the greater trochanter. The range of motion of the right hip joint was markedly restricted, with internal rotation limited to 30°, external rotation reaching 60°, abduction restricted to 45°, and adduction limited to 10°. In contrast, the left hip joint demonstrated normal mobility (flexion 140°, extension 30°, internal rotation 40°, external rotation 40°, abduction 40°, adduction 25°). A right pelvic tilt was observed, and both the Allis sign and right Thomas sign were positive. Preoperative laboratory results: White blood cells 4.53  ×  10⁹/L, CRP 3.57 mg/L, ESR 23 mm/h.

**Figure 1:**
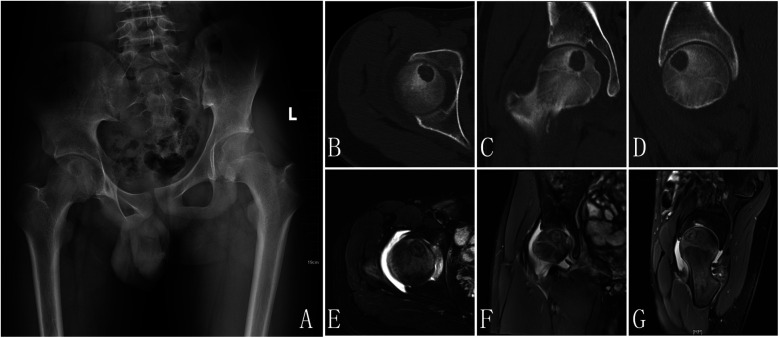
X-ray pre-op: a slightly hypoattenuating shadow was observed in the region of the right femoral head **(A)**, CT: a well-defined, round osteolytic lesion is identified within the right femoral head on coronal imaging. It demonstrates mild hyperdensity with a surrounding sclerotic margin **(B–D)**, MRI: a patchy area of mixed slightly high signal intensity is noted in the right femoral head, with clear margins. Patchy areas of high signal intensity are also present on PDWI in the right femoral head-neck junction and acetabulum. The synovium of the right femoral head is thickened, with associated joint effusion. Additionally, patchy high-signal-intensity regions are observed in the surrounding soft tissues on PDWI **(E–G)**.

Radiology examination: (1) x-ray: A roundish, slightly hypoattenuating shadow measuring approximately 17 mm in diameter is observed in the region of the right femoral head (Figure 1A) CT: A well-defined, round osteolytic lesion measuring approximately 12 mm  ×  16 mm is identified within the right femoral head on coronal imaging. It demonstrates mild hyperdensity with a surrounding sclerotic margin (Figures 1B–D) MRI: On T1WI and T2WI, a patchy area of mixed slightly high signal intensity is noted in the right femoral head, with clear margins and dimensions of approximately 12 mm  ×  16 mm. Patchy areas of high signal intensity are also present on PDWI in the right femoral head-neck junction and acetabulum. The synovium of the right femoral head is thickened, with associated joint effusion. Additionally, patchy high-signal-intensity regions are observed in the surrounding soft tissues on PDWI (Figures 1E–G).

**Figure 2:**
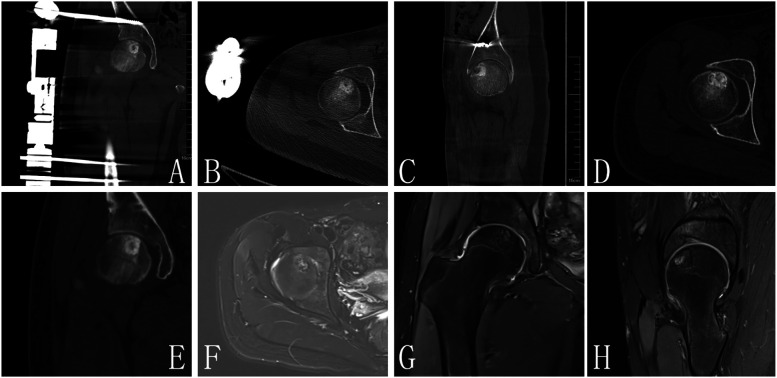
**(A–C)** Are postoperative images, CT **(D,E)** and MRI **(F–H)** of 1 year FU demonstrated no evidence of lesion recurrence in the femoral head, with radiological signs indicating complete resolution of the previous lesion.

**Figure 3:**
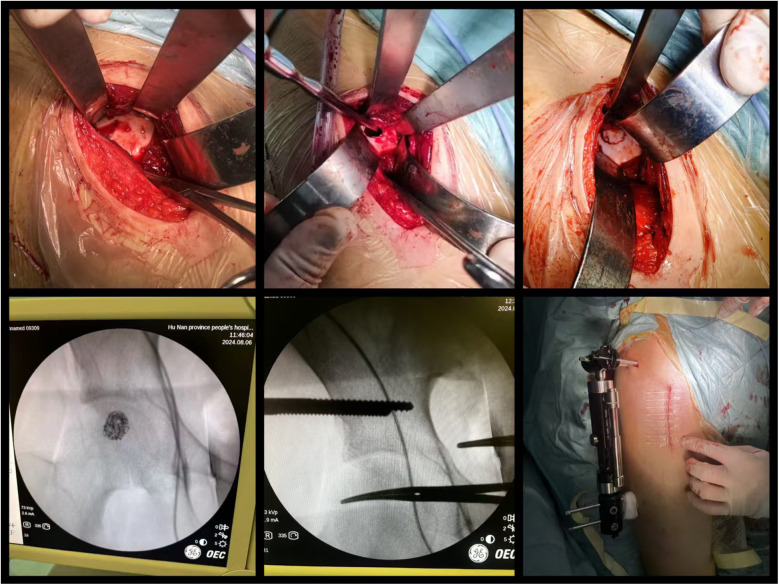
Intraoperative images.

Biopsy examination confirmed a chondroblastic mesenchymal tumor in the right femoral head lesion. Based on the patient's clinical history, physical findings,aforementioned imaging studies, and biopsy result, the preoperative diagnosis was formulated as Bone lesion in the right femoral head of uncertain nature: (1) Chondroblastoma, (2) Right hip joint synovitis. Following completion of preoperative preparation, a direct anterior approach (DAA) was performed to access the right hip joint, with an approximately 8 cm longitudinal incision. After skin incision was made, the lateral femoral cutaneous nerve was carefully dissected and preserved. The surgical plane between the sartorius and tensor fascia lata muscles was developed, and the rectus femoris muscle was gently retracted medially to expose the joint capsule. A T-shaped capsulotomy and trapdoor was performed, the cartilage above the lesion of femoral head was designed a round door, the posterior quarter of the round door was preserved as a hinge of the trapdoor. Intraoperative manipulation revealed a round osteolytic lesion within the femoral head measuring approximately 1.5 cm  ×  1.5 cm Viable lesion tissue was identified and thoroughly curetted (Figure 2). After complete debridement, allogeneic bone grafting was performed. Intraoperative C-arm fluoroscopy confirmed satisfactory placement of the bone graft. Two screws (5.0 mm × 150 mm) were then inserted into the ilium and two screws(5.0 mm × 150 mm) into femur respectively, and an external fixation frame was assembled(Carefix, Shanghai, China. http://www.carewayer.com/). Traction was applied to the femoral head to relieve intra-articular pressure and ensure joint stability. Once optimal alignment and tension were achieved, the external fixation frame was secured. We only did once traction during the entire treatment progress, the traction was done intra-operation and the distance is 5 mm. The wound was irrigated copiously, hemostasis was ensured, and the incision was closed in layers. Sterile dressings were applied postoperatively. Based on histomorphology, immunohistochemical profile (S-100(+), p63(partial +), CK(pan)(−), CK8/18(−), DOG1(partial +), Ki67(+, 5%), p53(scattered +), SATB2(partial +), MDM2(small foci +), CDK4(small foci +), H3.3G34W(−), H3K36M(+), Ki67(5%+), MDM2(osteoclast+), P53(10%+)), and clinical presentation, chondroblastoma was diagnosed (Figure 3). As the purpose of the arthrodiastasis is to relieve intra-articular pressure and ensure joint stability, we did NOT set a articulated hinge for the hip joint but just a fixation. The patient was asked non-weightbearing until the external fixation was removed. The external fixation frame was removed after a two-month follow-up period to adequately alleviate pressure on the femoral head.

Following removal of the external fixation frame, the patient underwent a two-week rehabilitation program focusing on hip joint functional exercises. The range of motion of the hip joint gradually returned to normal. During the initial phase of rehabilitation, the pediatric patient exhibited preoperative-level range of motion due to pain and discomfort associated with the external fixation device. The family requested discharge from the hospital, and no further rehabilitation therapy was pursued.

**Figure 4:**
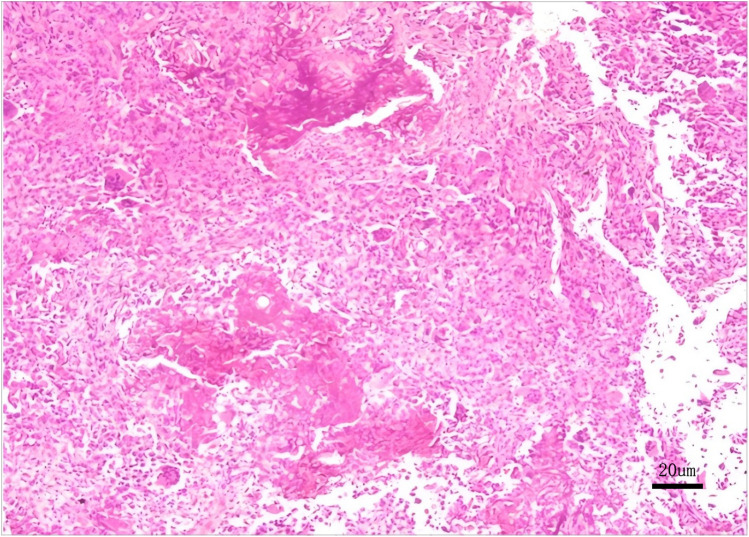
Histopathological biopsy reveals a proliferation of round to oval cells with scattered osteoclast-like multinucleated giant cells and characteristic chondroid matrix calcification, immunohistochemical findings certified chondroblastoma.

At the one-year follow-up, CT and MRI evaluations demonstrated no evidence of lesion recurrence in the femoral head, with radiological signs indicating complete resolution of the previous lesion (Figure 4). The bone density in the formerly affected area had normalized, confirming satisfactory osteogenesis.

## Discussion

Chondroblastoma constitutes approximately 1% of all primary bone tumors (2), and it predominantly occurs in adolescents and young adults, with a male-to-female ratio of approximately 2:1. The most frequently affected sites include the proximal tibia or femur, distal femur, and proximal humerus (3). As previously described, the case we report aligns with the typical presentation in terms of age, gender, and anatomical location. In contrast, chondroblastomas in elderly patients exhibit greater variability in localization and may involve non-tubular bones such as craniofacial or appendicular skeletal elements (e.g., hand and foot bones) (4, 5). Additionally, extremely rare cases have been reported in which the tumor arises within soft tissues adjacent to bone (6).

Chondroblastoma typically presents as a round or lobulated lesion with well-defined margins on gross examination. The cut surface is usually grayish-white, pale blue, or yellowish-brown, with a firm yet brittle consistency. Cystic changes, hemorrhage, and necrosis may be observed in certain areas. Microscopically, typical chondroblastoma exhibits characteristic features, including the presence of round or polygonal chondroblasts, multinucleated giant cells, and eosinophilic chondroid extracellular matrix, often accompanied by focal chondroid calcification (7). Pain is the primary clinical symptom of chondroblastoma and may worsen with physical activity. As the disease progresses, patients may develop local swelling, restricted joint movement, limping, and even pathological fractures. The patient described in this report presented with the commonly observed symptoms of right hip joint pain and limping. Although chondroblastoma is classified as a benign tumor, metastasi—most frequently to the lungs (8) -can occur, manifesting as palpitations, dry cough, dyspnea, and chest tightness (9). When patients present with such symptoms, thorough history-taking and physical examination are essential. Radiographic evaluation of the affected area should be the initial diagnostic step, typically revealing a well-defined, eccentric, oval or round osteolytic lesion with prominent marginal sclerosis involving the epiphysis. The lesion may appear hazy or mottled, with areas of sand-like calcifications (10). Computed tomography (CT) findings are generally consistent with those seen on plain radiographs but provide additional information regarding lesion size, internal structure, and anatomical relationships, aiding in differentiation from other entities such as giant cell tumor of bone and aneurysmal bone cyst (11). On MRI T1-weighted images, chondroblastoma typically demonstrates heterogeneous low signal intensity, while T2-weighted images may show regions of high signal intensity (12). Furthermore, MRI is particularly valuable for detecting joint effusion and perilesional bone marrow edema.

Chondroblastoma must be differentiated from several other bone tumors, with the following three being the most critical in differential diagnosis: (1) Giant cell tumor of bone (13): This lesion accounts for approximately 5% of all primary bone tumors and is more frequently observed in women aged 20–40. It typically occurs at the metaphyseal ends of long bones and presents clinically with localized pain. Histologically, it is characterized by numerous oval mononuclear cells resembling tumor cells and scattered osteoclast-like multinucleated giant cells. When chondroblastoma exhibits expansive growth without calcification, it may be misdiagnosed as a giant cell tumor of bone. (2) Aneurysmal bone cyst (14): This condition predominantly affects individuals aged 10–30 and is commonly found in long bones and vertebrae. Its pathological hallmark is the presence of blood-filled cavities separated by fibrous septa containing fibroblasts, monocytes, osteoclast-like giant cells, and reactive woven bone. (3) Chondromyxoid fibroma (15): This tumor primarily occurs in adolescents, with lesions typically located at the metaphyseal ends. Histologically, it is composed of immature myxoid mesenchymal tissue showing early signs of primitive cartilage differentiation. Additionally, areas of spindle or stellate-shaped cells within a myxoid matrix are characteristic features surrounding the lesion; such features are absent in chondroblastoma.

Surgery remains the primary treatment for chondroblastoma, and complete curettage of the lesion is essential for effective management (16–18). Following curettage, the defect can be filled with autologous bone graft, allogeneic bone graft, or bone cement (19, 20). Given that chondroblastoma commonly occurs in the epiphysis, the standard approach involving curettage and bone grafting may pose risks to the growth plate or articular cartilage, potentially leading to growth disturbances or secondary osteoarthritis (21). For this case, we did a preoperative biopsy, it was a percutaneous fine needle biopsy via guide by ultrasound. There is no doubt that the biopsy should be done pre-operation, the biopsy result determined the range of resection and detailed surgical procedures. This patient was finally managed using this curettage and bone grafting treatment strategy. We selected the direct anterior approach (DAA) for access to the right hip joint. This well-established surgical technique was originally described by Carl et al. and later popularized following modifications by Smith-Peterson. The DAA accesses the hip joint through the natural intermuscular plane between the tensor fascia lata and sartorius muscles, relying entirely on anatomical intervals without requiring muscle detachment. This results in minimal soft tissue trauma, reduced intraoperative bleeding, and accelerated postoperative recovery. However, meticulous dissection of the aforementioned muscular interval is crucial during the procedure; otherwise, it may cause iatrogenic injury to the surrounding musculature or the lateral femoral cutaneous nerve. Katagiri et al. (22) also employed this approach to treat chondroblastoma located in the femoral head, utilizing a trapdoor technique for lesion removal and achieving favorable outcomes. Recent studies have further confirmed the efficacy and safety of this surgical method (23). In contrast, the lateral trans-trochanteric approach offers a more limited surgical field, which increases the risk of incomplete resection, local recurrence (24), and damage to the growth plate (25), thereby affecting skeletal development in pediatric patients. Another alternative is the surgical dislocation approach, first introduced by Ganz et al. (26), which has been widely applied in the treatment of various pediatric hip disorders and benign tumors around the femoral head. Many surgeons prefer this approach for excising chondroblastomas due to its ability to preserve the medial circumflex femoral artery and provide excellent exposure of the femoral head (27, 28). Nevertheless, potential complications such as heterotopic ossification and nonunion at the osteotomy site remain important limitations of this technique.

As previously discussed, radical curettage for epiphyseal chondroblastoma frequently leads to complications such as osteoarthritis. Tumors located in the proximal femur appear particularly susceptible to postoperative secondary osteoarthritis and may even necessitate prosthetic joint replacement (29). Van Buul et al. (30) reported a case in which a child developed osteochondral defects 18 months after minimally invasive curettage and was subsequently treated with a partial joint surface replacement system. Francesco et al. (29) found that 12 out of 69 pediatric patients (17.5%) developed osteoarthritis following lesion curettage. Yang et al. (31) described a patient who experienced a femoral neck fracture three months after curettage combined with anhydrous ethanol-assisted treatment and bone grafting for femoral head chondroblastoma. These reports inevitably prompt us to consider how to minimize post-curettage complications and improve long-term outcomes and quality of life in children.

Arthrodiastasis was first introduced in 1979 for the treatment of Legg-Calvé-Perthes disease and has since been applied to various hip and knee joint disorders, including avascular necrosis of the femoral head, chondrolysis, and osteoarthritis, yielding favorable results (32–34). The theoretical foundation of this technique lies in the continuous distraction of the hip joint space, which reduces mechanical pressure on the femoral head and creates a conducive environment for healing and remodeling of the necrotic or surgically reconstructed femoral head. This mechanism is analogous to skeletal traction used in fracture management. Continuous arthrodiastasis can counteract compressive forces exerted by muscle contractions, reduce intra-articular inflammation, inhibit pathological bone remodeling, and promote a biological milieu favorable for cartilage repair and osseous regeneration (35).

External fixation technology has been widely utilized in the management of fractures, traumatic bone defects, deformity correction, and osteomyelitis, demonstrating consistent efficacy. Several studies have suggested its applicability in pediatric bone tumors (36) and for stabilizing complex defects following tumor resection (37). Kushwaha et al. reported successful use of external fixation combined with extensive curettage and bone grafting for treating aneurysmal bone cysts of the proximal femur (38), while others have documented its effectiveness in pathological fractures caused by non-ossifying fibroma (39). These findings provide valuable insights.

Although chondroblastoma is a benign tumor, when it involves the articular surface of the femoral head, postoperative structural support may be compromised, increasing the risk of articular surface collapse—a serious complication in growing children. To address this challenge, we employed an external fixation frame to maintain hip joint distraction, thereby preserving the integrity of the cartilage cap and preventing compression or collapse. Our clinical outcomes align with this rationale.

Maxwell et al. (40) reported complications including pin tract infection, joint stiffness, and bone pin fracture associated with the use of distraction arthroplasty in the treatment of Perthes disease. Aguado-Maestro et al. (41) emphasized that pin tract infection was the most frequently encountered complication. Cleary et al. (42) similarly observed transient pin tract infections when utilizing the Ilizarov frame for ankle distraction in pediatric patients with juvenile idiopathic arthritis. Therefore, careful attention must be given to postoperative pin tract care and structured rehabilitation programs to mitigate complications such as pin tract infection, joint stiffness, pain, discomfort, and external fixation-related issues—including loosening or fracture of bone pins. Incomplete debridement significantly increases the risk of local recurrence (43). Although adjuvant therapies such as radiofrequency ablation (44), cryotherapy (45), and hydrogen peroxide application (46) may carry a risk of additional growth plate injury, they have demonstrated certain efficacy in reducing recurrence rates. Huang et al. (47) noted that premature closure of the growth plate may occur following debridement surgery in skeletally immature patients. Interposition of polymethyl methacrylate (PMMA) has been suggested as an effective preventive strategy to preserve growth potential.

Furthermore, the use of a hinged external fixator for arthrodiastasis in treating hip disorders such as Perthes disease has also been proven effective. This approach maintains joint space while allowing a certain range of motion, which helps alleviate postoperative joint stiffness. However, several potential issues associated with the use of hinged external fixators must be addressed. For instance, improper positioning of the fixator hinge may lead to abnormal hip alignment. Additionally, the hinged external fixator requires more meticulous postoperative management, as it permits daily controlled hip movement and necessitates guided exercises under the supervision of a specialized rehabilitation physician (48, 49).

## Conclusion

The trapdoor technique allows for thorough curettage of lesions in femoral head chondroblastoma, while allogeneic bone grafting can achieve satisfactory osteogenesis. In this case, the combination of the trapdoor technique and arthrodiastasis effectively prevented chondral collapse of the femoral head during the critical period before bone graft integration and new bone formation occurred. Mid-term follow-up demonstrated favorable outcomes, supporting its consideration as a viable treatment option for femoral head chondroblastoma.

## Data Availability

The original contributions presented in the study are included in the article/Supplementary Material, further inquiries can be directed to the corresponding authors.
